# Multilevel Impacts of Transformational Leadership on Service Quality: Evidence From China

**DOI:** 10.3389/fpsyg.2019.01252

**Published:** 2019-05-30

**Authors:** Fangguo Su, Dejun Cheng, Shanshan Wen

**Affiliations:** ^1^College of Management, Shenzhen University, Shenzhen, China; ^2^School of Business, Nanjing University, Nanjing, China

**Keywords:** transformational leadership, affective commitment, psychological empowerment, service climate, service quality, multilevel analysis

## Abstract

In service contexts, leaders’ efforts to maintain and enhance employees’ service quality are vital to organizational performance. However, few studies have investigated the mechanisms underlying the relationship between transformational leadership (TFL) and employees’ service quality across levels. Based on the theory of planned behavior, this study investigated the multilevel impacts of TFL on employees’ service quality and the multilevel mechanisms mediating this relationship. Data were collected from 208 managers and 1,431 employees at 223 branches (chain restaurants) of a large catering corporation in southern China. The results showed that stronger TFL significantly improved employees’ service quality at both individual and branch level. Affective commitment and psychological empowerment partially mediated the relationship between individual-level TFL and employees’ service quality. Branch-level service climate fully mediated the relationship between branch-level TFL and employees’ service quality.

## Introduction

High-quality service is one of the core competitive advantages of service-based firms (Ehrhart et al., 2011). To achieve superior service quality, firms must ensure that their customers’ expectations are met and even exceeded. Positive interactions between employees and customers are essential to customer satisfaction (Liao and Chuang, 2004; Schneider et al., 2005). Frontline employees execute production and sales tasks simultaneously in the process of serving customers (Bowen and Waldman, 1999). Research has shown that transformational leadership (TFL) enhances service quality by stimulating employees’ enthusiasm and initiative (Liao and Chuang, 2007; Lee et al., 2011). Transformational leaders motivate their employees to achieve high performance by establishing an attractive and exciting vision, setting challenging yet achievable goals, being confident and optimistic and emphasizing team spirit and common values (Burns, 1978; Bass, 1985; Grant, 2012). Employees who work with transformational leaders are more likely to perceive their work as meaningful, have a higher level of commitment and are more psychologically empowered (Piccolo and Colquitt, 2006).

However, previous research has indicated that TFL has inconsistent effects on employees’ performance and attitudes toward their jobs (Barling et al., 1996; Bono and Judge, 2003; Li and Yuan, 2017). Employees may be unable to connect their leader’s vision and goals with their work circumstances (Grant, 2012). Alternatively, a compelling vision articulated by a leader may be perceived by employees as unrealistic or too difficult to accomplish. If employees are required to serve customers in their daily work, they may consider a leader’s vision to be more relevant and achievable. In this study, we therefore focused on the service industry, which requires employees to interact with customers in their daily work. This beneficiary contact is expected to strengthen the positive effects of TFL on employees’ performance (Grant, 2012). In short, employees may perceive their jobs as being more meaningful if they can help customers. Moreover, employees exhibit higher levels of commitment and psychological empowerment during face-to-face contact with customers than during other work tasks. Research has examined the cross-level effect of TFL on employees’ creativity (Jaiswal and Dhar, 2015; Bai et al., 2016), team creativity (Wang et al., 2016), employees’ service performance (e.g., Liao and Chuang, 2007) and organizational citizenship behaviors (Tse and Chiu, 2014). However, the multilevel effect of TFL on employees’ service quality and the multilevel mechanisms mediating this relationship have not been sufficiently studied (Su, 2013).

In this study, we investigated the effect of TFL on employees’ service quality at the individual and branch levels. At the branch level, TFL is expected to promote a positive service climate, which in turn improves employees’ service quality. At the individual level, employees who work with a transformational leader may be more committed to their roles and feel more empowered at work, which can help them to provide customers with a better service.

## Theory and Hypotheses

### Transformational Leadership (TFL)

According to Burns (1978), ‘leadership’ is defined as a leader’s ability to attract, motivate, stimulate and satisfy followers and help them to realize their common goals. Bass (1985) stated that transformational leaders focus on followers’ demands, motivate followers to realize the goals of the organization and encourage followers to pursue group interests and self-development. Recent research has found that mindfulness can positively influence TFL by fulfilling leaders’ need for autonomy, competence and relatedness (Decuypere et al., 2018). Transformational leaders achieve the aforementioned goals through their charisma or idealized influence, inspiration or inspirational motivation, intellectual stimulation and individualized consideration (Bass, 1985, 1995; Bass and Avolio, 1990). Leaders with charisma and idealized influence earn followers’ respect by setting good examples, and encourage subordinates to follow these examples through strong beliefs and high moral standards. In addition, leaders interact with followers to establish common goals that can shape and strengthen followers’ sense of mission. For example, transformational leaders who emphasize green (environmentally friendly) values may strengthen followers’ green identity by triggering value congruence (Wang et al., 2018). Through inspirational motivation, leaders encourage and guide followers to confront difficulties and challenges, realize their goals and meet high expectations. They seek to inspire followers through affective and hortatory statements. Leaders with these transformational capabilities are sensitive to environmental changes and aware of individual needs, enabling them to steer collaboration and partnerships in a global context (Kumar, 2019). Such leaders also engage in intellectual stimulation, encouraging followers to question the current situation and conventions, think independently and be actively creative. Through individualized consideration, transformational leaders tailor their guidance to followers’ particular characteristics and situations. For instance, a study conducted in Italy showed that leaders’ ability to understand followers’ stress is beneficial to followers’ mental health and psychological well-being (Giorgi et al., 2014).

### Service Quality

Service employees have a considerable influence on service quality. In contrast to goods, services are characterized by intangibility, heterogeneity and the synchronization of production and consumption (Parasuraman et al., 1985). Service quality as perceived by customers reflects customers’ overall judgment of the characteristics of the service provided (Zeithaml et al., 1988). Parasuraman et al. (1985) divided service quality as perceived by customers into 10 dimensions: reliability, responsiveness, competence, access, courtesy, communication, credibility, security, understanding/knowing and tangibility. In a later study, Parasuraman et al. (1988) analyzed service quality using the following five attributes: tangibility, reliability, responsiveness, assurance, and empathy.

### TFL and Service Quality

According to its scope of influence, TFL can be categorized as either individual-level or branch-level (Liao and Chuang, 2007). Individual-level TFL reflects individuals’ perceptions of their manager’s or supervisor’s leadership style. From the perspective of employees, transformational leaders set examples during management tasks, establish common goals, strengthen their sense of mission and provide encouragement. Individual-level TFL thus emphasizes individuals’ feelings about and subjective judgments of leadership style. Due to differences between individuals and in leader–member contact frequency, employees may interpret the same leadership style differently. Therefore, perceived leadership style may have different effects on employees’ attitudes toward work and the quality of service they provide (e.g., Liao and Chuang, 2007). Transformational leaders promote greater adherence to service quality guidelines to increase their firms’ customer service performance (Lam and Schaubroeck, 2000; Schaubroeck et al., 2016).

Branch-level TFL refers to the leadership of the entire working unit that forms the work context for individuals. Thus, branch-level TFL reflects the shared judgment of leadership style of all employees. Branch-level TFL motivates employees to improve service quality by fostering their aspiration to achieve shared common goals, and indicates the degree to which leaders participate in the transformation of an organization (Menges et al., 2011). The behaviors of employees within an organization are influenced by their individual characteristics and surrounding environment (Raudenbush and Bryk, 2002; Zhang et al., 2003). Therefore, TFL at the branch level, the overarching setting in which employees work, can affect employees’ behavior and work performance. Transformational leaders can improve service quality by formulating appropriate service quality standards, communicating and demonstrating these standards to employees, and rewarding employees for offering high service quality.

Both individual- and branch-level TFL can significantly improve employees’ service performance by enhancing their positive attitude toward providing services (Liao and Chuang, 2007). In retail banks, for example, team-level TFL has been shown to have a significantly positive effect on team service quality by cultivating a service-oriented climate (Liao and Chuang, 2007; Lee et al., 2011). Therefore, we hypothesize as follows:

*Hypothesis 1a:* Individual-level TFL and employees’ service quality are positively correlated.
*Hypothesis 1b:* Branch-level TFL and employees’ service quality are positively correlated.

### Effects of Individual-Level TFL on Employees’ Affective Commitment (Would Do) and Psychological Empowerment (Could Do)

According to the theory of planned behavior (Ajzen, 1991), whether one is willing to engage in a given behavior depends on one’s behavioral intentions. Three factors can predict one’s behavioral intentions: attitude toward the behavior, subjective norms and perceived behavioral control. In service settings, employees have the discretion to provide customers with low- or high-quality services. Based on the theory of planned behavior and previous research, transformational leaders can have a highly significant impact on employees’ work attitudes and organizational norms, which may then affect employees’ work behaviors.

Organizational commitment refers to employees’ psychological identification with and acceptance of their organization’s goals and values. Employees with high organizational commitment are willing to assume greater responsibility for their organization’s interests and make more effort to achieve its goals. Employees’ affective commitment, a type of organizational commitment, reflects their emotional attachment to the organization (Meyer et al., 1993). Employees who exhibit stronger affective commitment to their organization invest greater effort in their work and are willing to assume more responsibility. TFL can strengthen employees’ organizational commitment (Avolio et al., 2004; Chen et al., 2006), because transformational leaders can motivate employees to pursue goals and values congruent with those of the organization. Employees with high levels of commitment and satisfaction provide superior services to customers. In short, when individual employees perceive their leaders to be transformational, they are likely to deliver high-quality services, due to the positive attitudes fostered by their leaders. Affective commitment thus reflects what employees *would do* for the organization.

Psychological empowerment is a motivational construct with four dimensions: meaning, self-efficacy, self-determination and impact (Thomas and Velthouse, 1990; Spreitzer, 1995). ‘Meaning’ refers to whether work goals or purposes are consistent with an individual’s work beliefs, values and behaviors. Self-efficacy represents an individual’s belief in their ability to successfully complete tasks or activities (Bandura, 1986). Individuals with high levels of self-determination in a work setting independently choose to perform or change their actions and determine their own work practices, processes and schedules. ‘Impact’ refers to an individual’s influence on their organization’s strategy, administrative system and professional outcomes. Studies have discovered a significantly positive correlation between TFL and psychological empowerment (Avolio et al., 2004; Chen et al., 2006; Barroso Castro et al., 2008; Gumusluoglu and Ilsev, 2009; Allameha et al., 2012). Transformational leaders accelerate their followers’ growth and foster their independence and psychological empowerment (Bass, 1985; Dvir et al., 2002; Kark et al., 2003). Compared with leadership based on power, duty and restricted levels of initiative, charismatic leadership improves the extent to which service employees anticipate and realize common work goals through effort and creativity at the service site. Employees who are more psychologically empowered exhibit and perceive greater control of their service behaviors and greater autonomy during the service process. Psychological empowerment thus reflects what employees *could do* for the organization.

Transformational leadership involves sharing common goals with employees, supporting employees, setting high service standards and expectations for employees and fostering positive service attitudes and actions among employees. Together, these activities constitute a basis for high service quality. If employees exhibit a positive attitude toward customer service but are not psychologically empowered, they cannot provide customers with high-quality services, despite understanding customers’ individualized demands. When employees perceive high levels of TFL, their psychological empowerment (*could do*) is high; therefore, they can implement and achieve high-quality standards. Psychological empowerment (*could do*) thus partially mediates the relationship between individual-level TFL and employees’ service quality.

Therefore, we propose the following hypotheses:

*Hypothesis 2a*: Individual-level affective commitment partially mediates the relationship between individual-level TFL and employees’ service quality.
*Hypothesis 2b*: Individual-level psychological empowerment partially mediates the relationship between individual-level TFL and employees’ service quality.

### Branch-Level TFL: Transforming the Service Climate (Should Do)

The service climate has been defined as ‘employees’ perceptions of the practices, procedures, and behaviors that get rewarded, supported, and expected with regard to customer service and customer service quality’ (Schneider et al., 1998, p. 151). In other words, the service climate reflects employees’ general perceptions of organizational incentives and support, employees’ service orientation, and the organization’s service policies, procedures and standards of service quality. At branch level, transformational leaders may play an important role in shaping a company’s service climate (Liao and Chuang, 2007). Schneider et al. (1998) suggested that transformational leaders appreciate and reward employees’ excellent service behavior, encourage and help employees to provide customers with high-quality and efficient services, and promote the concept of service orientation. Accordingly, TFL assists in shaping employees’ perceptions of a customer-oriented service climate within a branch. Hong et al. (2013) highlighted that leadership positively affects service climate. At branch level, therefore, TFL may strengthen employees’ perceptions of the overall positive service climate of a branch.

**FIGURE 1:**
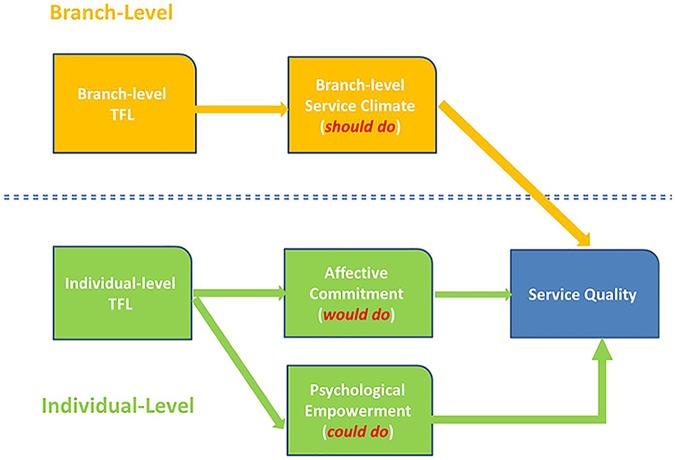
Multilevel model of transformational leadership and service quality.

Employees who work in organizations with a stronger service climate are more likely to provide high service quality (Hui et al., 2007). Based on the theory of planned behavior, when employees internalize service-oriented norms, they are more likely to deliver high-quality services to their customers. Branch-level service climate reflects employees’ common cognition of the service-oriented atmosphere within a branch. Therefore, a strong service climate at branch level typically creates an invisible service quality standard. Liao and Chuang (2007) demonstrated that branch-level TFL significantly enhances employee service performance by creating a strong service climate. Employees with strong perceptions of branch-level TFL believe that everyone in the branch should exert considerable effort to achieve high service quality. Service climate therefore determines employees’ perceptions of what they *should do* for the organization (Please refer to Figure 1 for the conceptual model).

Thus, we propose the following hypothesis:

*Hypothesis 3:* Branch-level service climate mediates the relationship between branch-level TFL and employee service quality.

## Materials and Methods

### Sample and Procedure

We selected 223 branches (chain restaurants) of a large catering corporation in southern China. Each branch had approximately 60 employees. Research assistants and coordinators were responsible for delivering and collecting the questionnaires. We gave them instructions regarding the requirements and procedures of administering the questionnaires in each branch. The questionnaires were anonymous to ensure that the respondents felt able to freely express their thoughts. To ensure objectivity and independence, we provided a separate envelope for each questionnaire; respondents placed their completed questionnaires in the envelopes and then returned them to our collectors. We randomly selected 30 employees from each of five branches, and randomly selected 15 employees from each of the remaining 218 branches. In addition, one manager/leader was selected from every branch. We delivered 3,420 questionnaires to the employees and collected 1,960, yielding a response rate of 57.3%. We then removed questionnaires with incomplete and/or inappropriate answers, leaving 1,690 valid questionnaires (valid response rate: 49.4%). As branch-level TFL and branch-level service climate were aggregated by individual variables, we ensured that the number of participants for each branch was no smaller than 10% of the total number of employees at that branch by excluding branches with fewer than six participants (approximately 10%). We thus removed a further 259 employee questionnaires, yielding an employee sample of 1,431 (these employees were in 154 branches). We deliver 223 branch manager questionnaires, of which 220 were collected, giving a response rate of 98.7%. We removed questionnaires with incomplete and/or inappropriate answers, resulting in 208 valid questionnaires (valid response rate: 93.3%). As shown in Table 1, the sample consisted of participants across different categories of employees: age, gender, education level and tenure. Of the employees, 85.6% were female, 93.1% were aged <31, 41.1% had graduated from high school and 70.8% had worked at their respective branches for more than 6 months. To increase the validity of the analysis by accounting for the sample characteristics, we controlled for employees’ gender, age, education level, and tenure.

**Table 1:** Data sample characteristics of employees.

		Frequency	Percent (%)
Gender	Male	206	14.4
	Female	1225	85.6
Age	18–24	951	66.5
	25–30	381	26.6
	31–34	65	4.5
	35–40	28	2.0
	41 years old or older	6	0.4
Education	Junior high school and below	208	14.5
	High school	588	41.1
	Junior college	301	21.0
	Undergraduate	270	18.9
	Master’s degree	63	4.4
	Doctorate	1	0.1
Tenure^∗^	6 months or less	418	29.2
	6 months–1 year	431	30.2
	1–3 years	431	30.1
	4–5 years	111	7.8
	6–8 years	33	2.3
	8 years or more	6	0.4

*N = 1431. ^∗^Tenure in this branch has one missing data.*

To cross-validate the analysis based on the above sample, we also asked managers to rate the branch-level TFL and did robustness test later on. We matched the branch-level TFL data rated by managers with the employee-level data. As a result, 140 employee questionnaires and 70 manager questionnaires were excluded. Sample characteristics of managers are showed in Table 2. Of the branch managers, 52.2% were female, 59. 4% were aged 25–30, 39.1% had graduated from university and 72.5% had been employed at the company for more than 5 years. The sample for robustness test comprised 138 valid questionnaires completed by managers and 1,291 valid questionnaires completed by employees.

**Table 2:** Data sample characteristics of managers.

		Frequency	Percent (%)
Gender	Male	66	47.8
	Female	72	52.2
Age	<24	0	0
	25–30	82	59.4
	31–34	40	29.0
	35–40	16	11.6
	>41	0	0
Education	Junior college and below	84	60.9
	Undergraduate	54	39.1
	Master’s degree	0	0
	Doctorate	0	0
Tenure	6 months or less	1	0.7
	6 months to 1 year	0	0
	1–3 years	5	3.6
	4–5 years	32	23.2
	6 years or more	100	72.5

*N = 138.*

### Measures

We used the existing Chinese version of the TFL scale developed by Chen et al. (2006), but translated the other scales into Chinese from their English versions through a translation–back translation procedure (Brislin, 1980). All of the items were rated on a five-point Likert scale, ranging from 1 (strongly disagree) to 5 (strongly agree).

#### Individual-Level TFL

Combining the scale developed by Waldman et al. (2001) with the multifactor leadership questionnaire developed by Bass (1985) and Bass and Avolio (1990), Chen et al. (2006) constructed a TFL scale and tested it in the Chinese context. We used the eight-item scale reported by Chen et al. (2006) to measure individual-level TFL. Cronbach’s alpha for the scale was 0.90. Confirmatory factor analysis (CFA) revealed that the TFL measure fitted our data well [χ^2^(13) = 30.19, RMSEA = 0.04, CFI = 0.99, AGFI = 0.98]. These results provided evidence of the construct validity of the TFL measure in the Chinese context.

#### Branch-Level TFL

We used two methods to measure branch-level TFL, as acquiring data from different sources allowed us to control for common method variance (Podsakoff et al., 2003) and test the robustness of the results. First, we conducted general multilevel evaluation using the ‘bottom-up’ data aggregation method (Kozlowski and Klein, 2000). We aggregated employees’ evaluations of branch managers’ TFL as branch-level TFL scores. Second, the branch managers completed the eight-item TFL scale reported in Chen et al. (2006).

#### Affective Commitment

We used Meyer et al. (1993) six-item scale to measure affective commitment. The results of factor analysis revealed that the factor loadings of two negatively worded items were less than 0.40. Therefore, these two items were deleted. Cronbach’s alpha for affective commitment was 0.82. The results of CFA of the factor structure for affective commitment [χ^2^(1) = 2.56, RMSEA = 0.03, CFI = 0.99, AGFI = 0.99] adequately corresponded with the sample data.

#### Psychological Empowerment

We used Spreitzer (1995) 12-item scale to measure psychological empowerment, comprising four factors: work meaning, self-efficacy, job autonomy and influence. Cronbach’s alpha was 0.89. The results of CFA indicated that the psychological empowerment measure showed a good fit to our sample data [χ^2^(44) = 129.44, RMSEA = 0.04, CFI = 0.99, AGFI = 0.97].

#### Branch-Level Service Climate

We adopted the seven-item service climate scale developed by Schneider et al. (1998). Cronbach’s alpha was 0.88. The results of CFA of the service climate scale indicated a good fit [χ^2^(9) = 24.49, RMSEA = 0.04, CFI = 0.99, AGFI = 0.98]. As an organization’s service climate represents employees’ common perceptions of the organization’s service practices, procedures and behaviors, based on the general multilevel evaluation process using the ‘bottom-up’ data aggregation method (Kozlowski and Klein, 2000), we aggregated individual employees’ service climate perceptions at the store level to measure the service climate.

#### Service Quality

Service quality was measured using the six-item Likert scale developed and adapted to the Chinese service industry by Ling and Wang (2009). The participating employees were asked to evaluate the service quality they provided. Sample questions include ‘I always work hard to improve my service quality,’ ‘I proactively help my customers’ and ‘I try my best to satisfy my customers.’ Cronbach’s alpha was 0.86. The results of CFA indicated that the service quality measure fitted our sample data well [χ^2^(3) = 2.22, RMSEA = 0.00, CFI = 0.99, AGFI = 0.99].

#### Control Variables

We controlled for the following variables: gender, age, education level, and tenure.

### Aggregation Tests

The within-group agreement statistics (*r*_wg_), i.e., average value and median, were 0.97 and 0.98 for the TFL scale and 0.96 and 0.97 for the service climate scale, respectively. The values for both exceeded the acceptability standard of 0.70 (James et al., 1984, 1993; Bliese, 2000). The intraclass correlation [ICC (1)] of the TFL scale was 0.24, which exceeded the standard of 0.12 (James, 1982), and its χ^2^(153) was 586.96 (*p* < 0.01). The ICC (1) index of the service climate scale was 0.26, which exceeded the standard, and its χ^2^(153) was 648.54 (*p* < 0.01). The reliability of the group mean [ICC (2)] of the TFL scale was 0.74, and the ICC (2) of the service climate scale was 0.76, both of which exceeded the value commonly considered the lowest acceptable, namely 0.50 (Klein et al., 2000). Therefore, the data aggregation test was sufficient to reveal intragroup consistency and aggregating individual-level TFL and branch-level service climate was reasonable and valid.

### Data Analysis

We conducted three-step hierarchical linear modeling (HLM) (using the HLM 6.02 software package) as suggested by Kreft and de Leeuw (1998), Raudenbush and Bryk (2002), and Liao and Chuang (2007), as follows. (1) We used a null model to evaluate whether employee service quality had significant interclass variance and whether cross-level testing was necessary. (2) We conducted first-level stepwise regression and introduced the control, independent and mediation variables sequentially. (3) If the interclass variance of employees’ service quality was significant at the individual level, the independent and mediation variables were introduced at the branch level.

## Results

### Construct Distinctiveness of the Study Variables

To examine the construct distinctiveness of the study variables, we conducted CFA of the four individual-level variables. As shown in Table 3, the fit indices revealed that the hypothesized four-factor model offered an adequate fit to the data (χ^2^ = 416.01, df = 90, TLI = 0.96, CFI = 0.97, and RMSEA = 0.05); it fitted the data better than any of the alternative nested models. This shows that the constructs used to measure the four individual-level variables (TFL, affective commitment, psychological empowerment, and service quality) were sufficiently distinct. Therefore, there was no serious common method bias in this study.

**Table 3:** Results of confirmatory factor analysis of the four individual-level variables.

Model	χ^2^	df	TLI	CFI	RMSEA	^Δ^χ^2^
Four-factor model	416.01	90	0.96	0.97	0.05	
Three-factor model: TFL and AC combined	1491.68	101	0.86	0.88	0.10	1075.67^∗∗^
Two-factor model: TFL, AC, and PE combined	1654.28	103	0.85	0.87	0.10	1238.27^∗∗^
One-factor model	2252.79	104	0.79	0.82	0.12	1836.78^∗∗^

*TFL, transformational leadership; AC, affective commitment; PE, psychological empowerment; SQ, service quality. ^∗∗^p < 0.01.*

### Descriptive Statistics of Variables

Table 4 presents Spearman correlations for the individual-level variables under study. The mean values and standard deviations of branch-level TFL were 4.47 and 0.38, respectively, and those of branch-level service climate were 4.30 and 0.43, respectively.

**Table 4:** Spearman correlations for the individual-level variables.

	Gender	Age	Education	Tenure	TFL	AC	PE	SQ
Gender								
Age	0.05^∗^							
Education	−0.11^∗∗^	−0.15^∗∗^						
Tenure	−0.01	0.32^∗∗^	−0.09^∗∗^					
TFL	−0.11^∗∗^	0.09^∗∗^	0.05^∗^	0.11^∗∗^	(0.90)			
AC	−0.06^∗^	0.11^∗∗^	0.05	0.14^∗∗^	0.58^∗∗^	(0.82)		
PE	−0.13^∗∗^	0.11^∗∗^	0.04	0.14^∗∗^	0.61^∗∗^	0.68^∗∗^	(0.89)	
SQ	−0.11^∗∗^	0.16^∗∗^	0.05	0.13^∗∗^	0.57^∗∗^	0.63^∗∗^	0.69^∗∗^	(0.86)

*^∗^p < 0.05, ^∗∗^p < 0.01 (two-tailed test). N = 1,431. TFL, transformational leadership; PE, psychological empowerment; AC, affective commitment; SQ, service quality. The internal consistency coefficients in parentheses are Cronbach’s alpha values on the diagonal.*

### Hypothesis Testing

Before conducting the HLM analysis, we set up a null model (see Table 5, Model 1) with no predictive variables to decompose the interclass and intraclass variance of service quality. The results revealed that service quality had an intraclass variance, σ^2^, of 0.33, and an interclass variance, τ_00_, of 0.06. ICC (1) = interclass variance τ_00_/(intraclass variance σ^2^+ interclass variance τ_00_) = 0.16, indicating that 16% of the variance in employees’ service quality derived from interclass variance and 84% from intraclass variance. The results of chi-square tests revealed that the interclass variance was significant: χ^2^(153) = 416.23 (*p* < 0.01). Thus, employee service quality was influenced by both individual-level and branch-level TFL, allowing multilevel analysis to be carried out with the dependent variable.

**Table 5:** Hierarchical linear modeling results.

Dependent variable		Service quality						AC	PE
		Model 1	Model 2	Model 3	Model 4	Model 5	Model 6	Model 7	Model 8
Intercept	γ_00_	4.44^∗∗^	4.41^∗∗^	4.43^∗∗^	4.37^∗∗^	4.36^∗∗^	4.36^∗∗^	4.26^∗∗^	4.25^∗∗^
Level 1: individual level									
Gender	γ_10_		−0.15^∗∗^	−0.12^∗∗^	−0.04	−0.03	−0.04	−0.07	−0.18^∗∗^
Age	γ_20_		0.13^∗∗^	0.10^∗∗^	0.08^∗∗^	0.08^∗∗^	0.08^∗∗^	0.04	0.05
Education	γ_30_		0.02	0.01	0.01	0.01	0.01	0.02	−0.00
Tenure	γ_40_		0.03	0.02	−0.00	−0.01	−0.00	0.06^∗∗^	0.04^∗^
L-1 TFL	γ_50_			0.48^∗∗^	0.19^∗∗^	0.19^∗∗^	0.19^∗∗^	0.58^∗∗^	0.50^∗∗^
L-1 AC	γ_60_				0.19^∗∗^	0.19^∗∗^	0.19^∗∗^		
L-1 PE	γ_70_				0.36^∗∗^	0.36^∗∗^	0.36^∗∗^		
Level 2: branch level									
L-2 TFL	γ_01_					0.57^∗∗^	0.07		
L-2 SC	γ_02_						0.53^∗∗^		
Total deviance		2638.39	2610.95	2249.98	1815.75	1716.18	1666.20	2770.43	2580.31

*L-1 TFL, individual-level TFL; L-1 AC, individual-level affective commitment; L-1 PE, individual-level psychological empowerment; L-2 TFL, branch-level TFL; L-2 SC, branch-level service climate. ^∗^p < 0.05, ^∗∗^p < 0.01; Coefficients are estimations of the fixed effects with robust standard errors. For level 1 (L-1), N = 1,431 for the individual-level variables. For level 2 (L-2), N = 154 for the branch-level variables.*

According to Hypothesis 1a, TFL has a positive influence on employees’ service quality. After the independent variable, individual-level TFL, was introduced, the robustness results (see Table 5, Model 3) revealed that the coefficient between TFL and employees’ service quality was 0.48 (*p* < 0.01), indicating a significantly positive correlation between TFL and employees’ service quality. The total deviance in Model 3 was 360.97 times smaller than that in Model 2. A smaller total deviance indicated a better model fit (Liao and Chuang, 2007). Therefore, Hypothesis 1a was supported.

Our tests of the multilevel mediation effect using the previously described procedures (Krull and MacKinnon, 2001; Mathieu and Taylor, 2007) generated the following results. The coefficient between TFL and affective commitment was 0.58 (*p* < 0.01) (see Table 5, Model 7). The coefficient between TFL and psychological empowerment was 0.50 (*p* < 0.01) (see Table 5, Model 8). The regression coefficient for affective commitment and service quality was 0.19 (*p* < 0.01) (see Table 5, Model 4). The regression coefficient for psychological empowerment and service quality was 0.36 (*p* < 0.01). In addition, the total deviance in Model 4 was 434.23 times smaller than that in Model 3. To more rigorously test the mediation effect, we followed Preacher et al. (2010) parametric bootstrapping method. The results of 20,000 Monte Carlo replications revealed a positive indirect relationship between TFL and service quality through affective commitment; the coefficient of the indirect effect was 0.11, with a 95% bias-corrected bootstrap confidence interval (CI) of [0.07, 0.15]. As zero was not included in the 95% CI, affective commitment partially mediated the relationship between TFL and service quality. Therefore, Hypothesis 2a was supported. Using the same parametric bootstrapping procedure (Preacher et al., 2010), we found that the coefficient of the indirect effect was 0.18, and the 95% bias-corrected bootstrap CI was [0.15, 0.22]. As zero was not included in the 95% confidence interval, we discovered a partially significant mediation effect of individual-level TFL on employee service quality through psychological empowerment. Therefore, Hypothesis 2b was supported.

To test Hypotheses 1b and 3, we introduced branch-level TFL. The coefficient between branch-level TFL and employees’ service quality was 0.57 (*p* < 0.01) (see Table 5, Model 5), indicating that branch-level TFL has a significantly positive influence on employees’ service quality. The total deviance in Model 5 was 99.57 times smaller than that in Model 4. Therefore, Hypothesis 1b was supported. The coefficient between branch-level TFL and service climate was 0.95 (*p* < 0.01). The regression coefficient for branch-level service climate and service quality was 0.53 (*p* < 0.01) (see Table 5, Model 6). The total deviance in Model 6 was 49.98 times smaller than that in Model 5. Using the same parametric bootstrapping procedure (Preacher et al., 2010), we found a positive indirect relationship between TFL and service quality through service climate. The coefficient of the indirect effect was 0.51 and the 95% bias-corrected bootstrap CI was [0.36, 0.67]. As zero was not included in the 95% CI, we concluded that branch-level service climate had a robust mediation effect on the relationship between branch-level TFL and employees’ service quality. In other words, service climate completely mediated the relationship between TFL and service quality. Therefore, Hypothesis 3 was supported.

### Robustness Test

To test the robustness of the multilevel model, we obtained TFL scores from managers’ self-ratings in the data acquisition process. We linked the managers’ self-rated TFL data at branch level with the corresponding employee data at each branch, and tested the link using multilevel models. The results of the HLM analysis supported all of the foregoing hypotheses (see Table 6). Therefore, we inferred that the results of the study were robust.

**Table 6:** Hierarchical linear modeling results (dependent variable: service quality).

		Model 1	Model 2	Model 3	Model 4	Model 5	Model 6
Intercept	γ_00_	4.43^∗∗^	4.41^∗∗^	4.45^∗∗^	4.39^∗∗^	4.38^∗∗^	4.39^∗∗^
Level 1: individual level							
Gender	γ_10_		−0.16^∗∗^	−0.13^∗∗^	−0.06	−0.06	−0.05
Age	γ_20_		0.13^∗∗^	0.11^∗∗^	0.09^∗∗^	0.09^∗∗^	0.08^∗∗^
Education	γ_30_		0.02	0.01	0.01	0.01	0.01
Tenure	γ_40_		0.04	0.02	−0.00	−0.00	−0.00
L-1 TFL	γ_50_			0.47^∗∗^	0.20^∗∗^	0.19^∗∗^	0.20^∗∗^
L-1 AC	γ_60_				0.18^∗∗^	0.18^∗∗^	0.18^∗∗^
L-1 PE	γ_70_				0.36^∗∗^	0.36^∗∗^	0.36^∗∗^
Level 2: branch level							
L-2 M-TFL	γ_01_					0.18^∗^	0.01
L-2 SC	γ_02_						0.59^∗∗^
Total deviance		2388.08	2357.14	2033.91	1644.82	1644.63	1514.06

*L-1 TFL, individual-level TFL; L-1 AC, individual-level affective commitment; L-1 PE, individual-level psychological empowerment; L-2 M-TFL, branch-level TFL rated by managers; L-2 SC, branch-level service climate. ^∗^p < 0.05, ^∗∗^p < 0.01. Coefficients are estimations of the fixed effects with robust standard errors. For level 1 (L-1), N = 1,291 for the individual-level variables. For level 2 (L-2), N = 138 for the branch-level variables.*

## Discussion

First, this study extended research on the relationship between TFL and employees’ service quality from a single level to multiple levels. Prior studies have focused on the single-level impact of TFL on service quality (e.g., Lee et al., 2011), whereas this study investigated individual- and branch-level TFL and found that both can significantly improve employees’ service quality. To improve employees’ service quality, TFL must be perceived both individually and collectively.

Second, this study used the overarching framework of the theory of planned behavior to elaborate the multilevel mechanisms through which the effects of individual- and branch-level TFL on service quality are mediated, namely service climate (*should do*), affective commitment (*would do*), and psychological empowerment (*could do*). When branch-level TFL is high, employees who perceive a strong service climate (*should do*) pursue the common goal of achieving high service quality. When employees perceive high levels of TFL, those with high organizational commitment (*would do*) invest considerable personal effort in delivering high service quality, and those with high perceived levels of psychological empowerment (*could do*) implement and achieve high quality standards.

This study revealed that individual-level TFL significantly improves employees’ service quality, and that this relationship is partially mediated by individual-level psychological empowerment. This finding is consistent with the results of Seibert et al. (2011). To increase service quality, service firms should enhance employees’ affective commitment and psychological empowerment. This study also revealed that branch-level service climate mediates the relationship between branch-level TFL and employees’ service quality. This is consistent with the finding of Hong et al. (2013) meta-analysis that leadership can have positive effects on service climate. Therefore, this study revealed the multilevel mediation mechanisms between TFL and employee service quality.

In the descriptive analysis, age, gender, and tenure were found to be significantly related to service quality, and educational level was positively related to TFL. Therefore, we added these four factors as control variables to the model analysis. The employees’ age was significantly related to their service quality, indicating that older employees are likely to deliver higher-quality services. This suggests that as employees become older, they accrue the experience, skills and strategies (e.g., emotion management) required to deliver high-quality services.

### Managerial Implications

Service enterprises should provide training and support for managers seeking to implement TFL. First, branch managers can set examples for followers by maintaining high standards for service quality and exhibiting other positive behavior to win followers’ respect and acknowledgment. Second, branch managers can use shared beliefs, emotional appeals and encouraging statements to motivate followers to achieve high service quality by overcoming difficulties and challenges and surpassing customer expectations. Third, branch managers can interact with their followers to establish high service quality standards for work groups. In the service industry, diverse customer demands and strict food monitoring regulations require managers to exhibit effective leadership and exert a powerful influence to overcome the external challenges associated with high service quality standards. Therefore, service firms can maintain a high service quality by implementing ongoing TFL training projects.

Service companies should also strengthen the branch-level service climate to help achieve a high service quality. Managers can strengthen employees’ perceptions of the meaningfulness of the work experience through rhetorical encouragement and practical action. They can improve employees’ self-efficacy through social cognition based methods, such as rewarding excellent employees. Managers can also encourage more employees to participate in the management decision-making process and propose new and improved methods for enterprise development, thereby expanding and strengthening employees’ influence. In addition, managers should seek to psychologically empower employees to encourage and equip them to provide flexible, prompt and proactive services for customers.

This study also showed that both individual- and team-level perceptions of TFL are important to the maintenance and improvement of employees’ service quality. Therefore, managers’ behavior and leadership styles should be consistent across contexts to persuade employees of the sincerity of their leaders’ vision and mission. Similarly, collective positive perceptions of TFL and the service climate should be built and sustained.

In today’s competitive and volatile economic environment, employees may suffer from job stress, which leads to mental and physical health problems (Giorgi et al., 2015; Mucci et al., 2016). To alleviate the negative impact of fear of economic crisis and other changes, leaders need to transform their organizations and employees by cultivating a service-oriented climate (branch level) that fosters employees’ affective commitment and psychological empowerment. In this way, transformational leaders can increase employees’ service quality.

### Limitations and Future Directions

This study had several limitations. First, the data on the primary independent and dependent variables in the model were self-reported. This may have been especially problematic for the measure of employees’ service quality. Critics may argue that we should have used customer-rated service quality. However, the sample consisted of service employees in restaurants, where a pseudo-relationship between customer and clients is common (Liao and Chuang, 2007). Customers may make limited effort to observe a particular employee’s service. Therefore, employees were assumed to possess the best knowledge of how their jobs are performed. Our CFA revealed that the one-factor model showed a poor fit to the data. Therefore, common method variance is unlikely to have significantly affected our findings. Future researchers should try to obtain ratings of employees’ service quality from multiple groups of raters, such as employees, their supervisors, and customers. Second, as all of the variables were assessed by the employees at a single time point, it was difficult to draw causal inferences. We collected the data from more than 200 branches, which required a lot of coordination and cooperation. Future studies should use a longitudinal design to explore how the observed relationships develop over time. Third, the data used in this study were collected from stores belonging to the same catering chain in southern China. To confirm the generalizability of the findings, future studies could collect data from other service settings. Nevertheless, the results of this research are not likely to be sample specific.

## Conclusion

Our results demonstrated that both individual- and branch-level TFL can significantly improve employee service quality. Affective commitment (*would do*) and psychological empowerment (*could do*) partially mediated the relationship between individual-level TFL and employees’ service quality. Branch-level service climate (*should do*) completely mediated the relationship between branch-level TFL and employees’ service quality. Based on the theory of planned behavior, we suggested and tested several key factors that affect employees’ service quality. However, service quality may also be affected by factors such as individual ability, personality and task characteristics. Future research could thus explore the potential influence of contextual factors on the relationships between individual-level and branch-level TFL and employee service quality. We hope that this study will stimulate more research on the multilevel relationship between TFL and employee service quality.

In conclusion, based on the theory of planned behavior, we demonstrated that employees’ service quality is significantly influenced by both individual- and branch-level TFL through multilevel mediating mechanisms, such as increasing employees’ affective commitment (*would do*) and psychological empowerment (*could do*) and strengthening the branch service climate (*should do*).

## Data Availability

All datasets generated for this study are included in the manuscript and/or the supplementary files.

## Ethics Statement

This study was carried out in accordance with the ethical standards of an institutional and/or a national research committee and with the 1964 Helsinki Declaration and its later amendments or comparable ethical standards. Prior to the research, ethical approval was obtained from the Academic Ethics Committee of Shenzhen University. Every participant was required to read and sign an informed consent form before participating in the research.

## Author Contributions

FS proposed the research idea, designed the study, collected and analyzed the data, and wrote the manuscript. DC collected the data and reviewed the manuscript. SW wrote, revised and critically reviewed the manuscript.

## Conflict of Interest Statement

The authors declare that the research was conducted in the absence of any commercial or financial relationships that could be construed as a potential conflict of interest.
